# Specific biomarkers for *C9orf72* FTD/ALS could expedite the journey towards effective therapies

**DOI:** 10.15252/emmm.201707848

**Published:** 2017-05-22

**Authors:** Rubika Balendra, Thomas G Moens, Adrian M Isaacs

**Affiliations:** ^1^ Department of Neurodegenerative Disease UCL Institute of Neurology London UK; ^2^ Department of Genetics, Evolution and Environment Institute of Healthy Ageing University College London London UK

**Keywords:** Biomarkers & Diagnostic Imaging, Genetics, Gene Therapy & Genetic Disease, Neuroscience

## Abstract

A hexanucleotide repeat expansion in the *C9orf72* gene is a common genetic cause of ALS and FTD. The repeats are translated into five different dipeptide repeat proteins (DPRs). In this issue, Lehmer *et al* (2017) demonstrate that one of these DPRs, poly(GP), can be measured in the CSF of individuals with *C9orf72* mutations. In conjunction with the findings from another recent study (Gendron *et al*, 2017), these DPR biomarkers may prove to be extremely valuable in the quest for effective therapies for C9FTD/ALS.

The discovery that GGGGCC expansions in the *C9orf72* gene are the most common genetic cause of amyotrophic lateral sclerosis (ALS) and frontotemporal dementia (FTD), collectively termed C9FTD/ALS, has transformed our understanding of these diseases (Rohrer *et al*, 2015). Proposed pathogenic mechanisms include loss of function of C9orf72 protein, and two gain‐of‐function mechanisms: toxicity from *C9orf72* repeat RNA or from DPRs produced by repeat‐associated non‐ATG translation, of which there are five, poly(GA), poly(GP), poly(GR), poly(PR) and poly(PA). There has been rapid translation of our understanding of the disease towards developing new therapies, especially for antisense oligonucleotides (ASOs), with human trials planned shortly. ASOs target *C9orf72* repeat RNA and can mitigate several *C9orf72* repeat‐induced pathologies both *in vitro* and *in vivo* (Zhang *et al*, 2015; Jiang *et al*, 2016; Gendron *et al*, 2017).

Although there is great excitement at the prospect of imminent clinical trials, it is critical we apply the lessons from past failures. In ALS, despite numerous Phase II/III clinical trials of potential therapeutics, only one drug, riluzole, has been shown to have a minimal effect on prolonging survival. The reasons for this are multifactorial (Mitsumoto *et al*, 2014), but one key factor that will facilitate better studies is demonstrating that biological effects are dependent on target engagement in appropriate and robust preclinical models and in subsequent clinical trials. This could be achieved by incorporating validated pharmacodynamic biomarkers, which demonstrate a biological effect in response to a therapeutic intervention, into preclinical and human studies. Importantly, an international effort to update the Guidelines for Clinical Trials in ALS to reflect these concepts is currently underway, with publication of the recommendations expected shortly.

Two key studies, one presented in this issue (Lehmer *et al*, 2017) and another recent study (Gendron *et al*, 2017), offer the tantalising prospect of such a biomarker for C9FTD/ALS. Building upon a previous study in a smaller cohort (Su *et al*, 2014), they validate that CSF poly(GP) is a sensitive and specific biomarker of *C9orf72* expansions using Meso Scale Discovery immunoassays, in both asymptomatic and symptomatic individuals.

In cellular and animal models, poly(GR) and poly(PR) are the most toxic DPRs, with poly(GA) also leading to some toxicity. However, both studies chose to measure poly(GP) for several reasons: (i) poly(GP) is one of the most frequent DPRs in C9FTD/ALS central nervous system (CNS) tissue; (ii) it is more soluble than poly(GA), which is the most frequent DPR; (iii) it appears to be a highly stable DPR, as demonstrated by its persistence in cultured iPSC‐derived neurons (Gendron *et al*, 2017); and (iv) it is produced from both sense and antisense *C9orf72* transcripts, although it is worth noting that ASO studies suggest sense transcripts are the major origin.

Interestingly, both studies show that CSF poly(GP) levels are comparable in asymptomatic and symptomatic individuals, although Gendron *et al* (2017) find a non‐significant trend towards an increase in symptomatic individuals. Further large‐scale, longitudinal studies will help determine if levels increase with symptom onset. Detection of poly(GP) in asymptomatic mutation carriers is in line with pathology studies showing CNS DPR inclusions can be present prior to symptom onset, and suggests that poly(GP) is being released from living neurons, rather than those undergoing neurodegeneration. Neither study finds an increase in CSF poly(GP) with disease progression, either measured longitudinally, or using clinical scores such as the revised ALS Functional Rating Scale and FTLD‐specific Clinical Dementia Rating, or an association with an ALS or FTD phenotype, age of disease onset, or survival. This stability of poly(GP) levels may enhance its value as a pharmacodynamic biomarker. In a clinical trial, for each patient, relative pre‐ and post‐intervention values would be compared; therefore, a stable marker showing normalisation with a therapy is ideal. As an elegant proof of principle of this concept, Gendron *et al* (2017) show that treating *C9orf72* iPSC‐derived neurons and a *C9orf72* mouse model with ASOs reduces poly(GP) levels. The levels of intracellular poly(GP) in *C9orf72* iPSC neurons correlates with extracellular levels in media bathing the cells, and in a mouse model, CSF poly(GP) correlates with poly(GP) in brain homogenates. This indicates that CSF poly(GP) levels are likely to reflect intraneuronal levels in the CNS. However, it is important to note that in their longitudinal analysis of a small patient cohort, in some individuals CSF poly(GP) naturally decreases over time, which should be accounted for in clinical trials. An emerging theme from trials in Alzheimer's disease (AD) is that administering treatment early in the disease course, before extensive neurodegeneration, may be most effective; therefore, detection of CSF poly(GP) in asymptomatic individuals may have great value for implementing early treatment. Lehmer *et al* (2017) show that CSF levels of neurofilaments (Nfl and pNfH), markers of axonal damage, are raised in symptomatic but not asymptomatic *C9orf72* mutation carriers, in agreement with recent studies of neurofilaments as ALS diagnostic and prognostic biomarkers (Fig 1). The combination of both biomarkers could provide evidence of target engagement and functional rescue.

**Figure 1:**
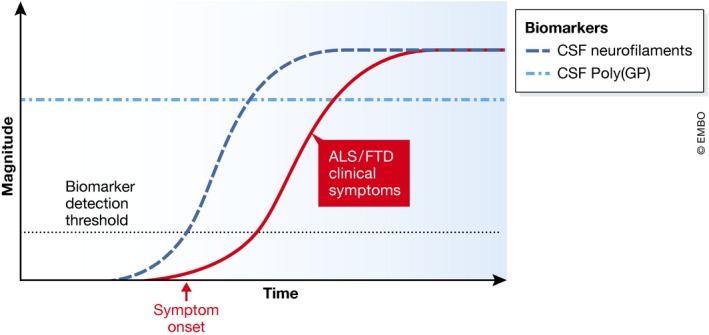
C9FTD/ALS CSF biomarkers CSF poly(GP) is similar in asymptomatic and symptomatic individuals, whilst CSF neurofilaments are detected in symptomatic individuals.

Lehmer *et al* (2017) suggest CSF poly(GP) could be used alongside genotyping as a diagnostic biomarker, and were able to reclassify one patient misdiagnosed with AD as C9FTD. Due to somatic instability of GGGGCC repeats, it is possible that patients without an expansion in blood DNA have expansions in the CNS; therefore, the addition of CSF poly(GP) could add diagnostic value. However, it is important to note that both studies describe a small number of false positives, non‐*C9orf72* individuals with elevated poly(GP), or false negatives, *C9orf72* individuals with non‐detectable poly(GP). Improving the sensitivity and specificity of these assays is critical and may require the use of other potentially more sensitive platforms, such as the Simoa (single molecule array), which was used on a subset of samples by Gendron *et al* (2017). Lehmer *et al* (2017) use monoclonal antibodies in their immunoassay, which are less variable and can be produced at higher throughput than polyclonal antibodies, so may provide an additional advantage. Developing similar assays for other more toxic DPRs may also be beneficial, as they may correlate better with progression or prognosis.

In addition to CSF, Gendron *et al* (2017) specifically detected poly(GP) in peripheral blood mononuclear cells. Detection in blood would be advantageous for repeat measurements in clinical trials. This could mark a significant step forward, but further studies are needed to determine whether blood and CSF poly(GP) levels correlate.

In conclusion, developing promising biomarkers should be integral to clinical trials in C9FTD/ALS, and a biomarker assay should ideally be sensitive, specific, reproducible with low variability, standardised and affordable. These studies now highlight CSF poly(GP) as one such potential candidate.
